# Reconstruction of a neglected extensor hallucis tendon rupture with an iliotibial band autologous graft: case report

**DOI:** 10.1097/RC9.0000000000000886

**Published:** 2026-08-24

**Authors:** Yusufa Fil Ardy, Muhammad Bayu Zohari Hutagalung, Andre Triadi Desnantyo

**Affiliations:** a Department of Orthopaedics and Traumatology, Faculty of Medicine, Universitas Airlangga, Surabaya, Indonesia; b Department of Orthopaedics and Traumatology, Dr. Soetomo General Academic Hospital, Surabaya, Indonesia

**Keywords:** extensor hallucis rupture, iliotibial band, Pulvertaft, tendon graft

## Abstract

**Background:**

Injury to the lower extremity, especially rupture of the extensor hallucis longus (EHL) tendon, is relatively rare. This case report presents a neglected case of EHL tendon rupture with a 10 cm gap, treated with a novel graft source – the iliotibial band (ITB) – using the Pulvertaft technique.

**Case report:**

A 34-year-old man presented with a previous rupture of the EHL tendon of his right great toe, with a 10 cm gap, 12 months before admission, for which a primary repair had already been performed immediately after the trauma. The pain persisted even 6 weeks post-surgery; therefore, after clinical and ultrasound evaluation, we found a rupture of the EHL tendon with the gap filled with fibrotic tissue. We performed a tendon graft for EHL reconstruction using the ITB with the Pulvertaft technique, which showed a good postoperative result.

**Discussion:**

Rupture of the EHL has proved to be troublesome, and the management of this case can be performed by direct repair in the acute setting or by tendon transfer or tendon graft. It should be performed properly to restore function.

**Conclusion:**

After the tendon grafting was performed, intraoperative evaluation found that there was no lag in the gliding of the tendon as it passed through the extensor retinaculum, and the excursion of the tendon was preserved.

## Introduction and importance

Extensor hallucis longus (EHL) rupture associated with trauma is relatively rare, and it is mostly caused by laceration from a small object. The true incidence and causes of EHL tendon lacerations are unknown; previous research on commonly injured tendons and muscles shows that 11% of all lower extremity cases involve the toe extensors^[^1–3^]^. Because injury to the EHL disrupts tendon integrity, in the long term it may result in deformities such as dorsal bunion, hallux flexus, hallux malleus, and, in neuropathic individuals[4]. A previous study showed that, of 16 patients with EHL rupture, 14 underwent direct repair and temporary fixation with a K-wire within 24 hours of injury, 1 was treated with a tendon graft, and 2 cases with partial EHL rupture were treated conservatively; all showed good results, as the patients were able to return to work 2.5–5 months after injury[5].

HIGHLIGHTS

- Rupture of the extensor hallucis tendon is uncommon.
- These injuries are commonly caused by direct trauma from a sharp object.
- Common management includes primary tendon repair and tendon grafting.
- Tendon grafting with fascia can be used to bridge the gap between the ends of a ruptured tendon.
- Postoperative treatment with a protective device and early mobilization shows good results.

Surgical intervention is needed to manage an EHL tendon rupture. The surgical treatment includes acute repair of the tendon rupture with a Krackow suture, which is recommended if performed within the first 24 hours after the injury, or tendon reconstruction using a graft. Graft options include the semitendinosus, extensor hallucis brevis, and peroneus longus tendons^[^5–7^]^. In most cases, the laceration is oriented horizontally and extended into a Z incision to allow access to both the proximal and distal stumps. The repair is performed directly–approximated with two locking sutures on both stumps– or using an autologous tendon graft or a tendon transfer. It is recommended to temporarily immobilize the patient to hold the metatarsophalangeal and interphalangeal joints with a short-leg splint for the initial 3 weeks to prevent potential stress on the recently reconstructed tendon. Physical training for gait, strength, and range of motion (ROM) should begin as soon as possible within the first 6 weeks[1].

## Case report

**Figure 1.**
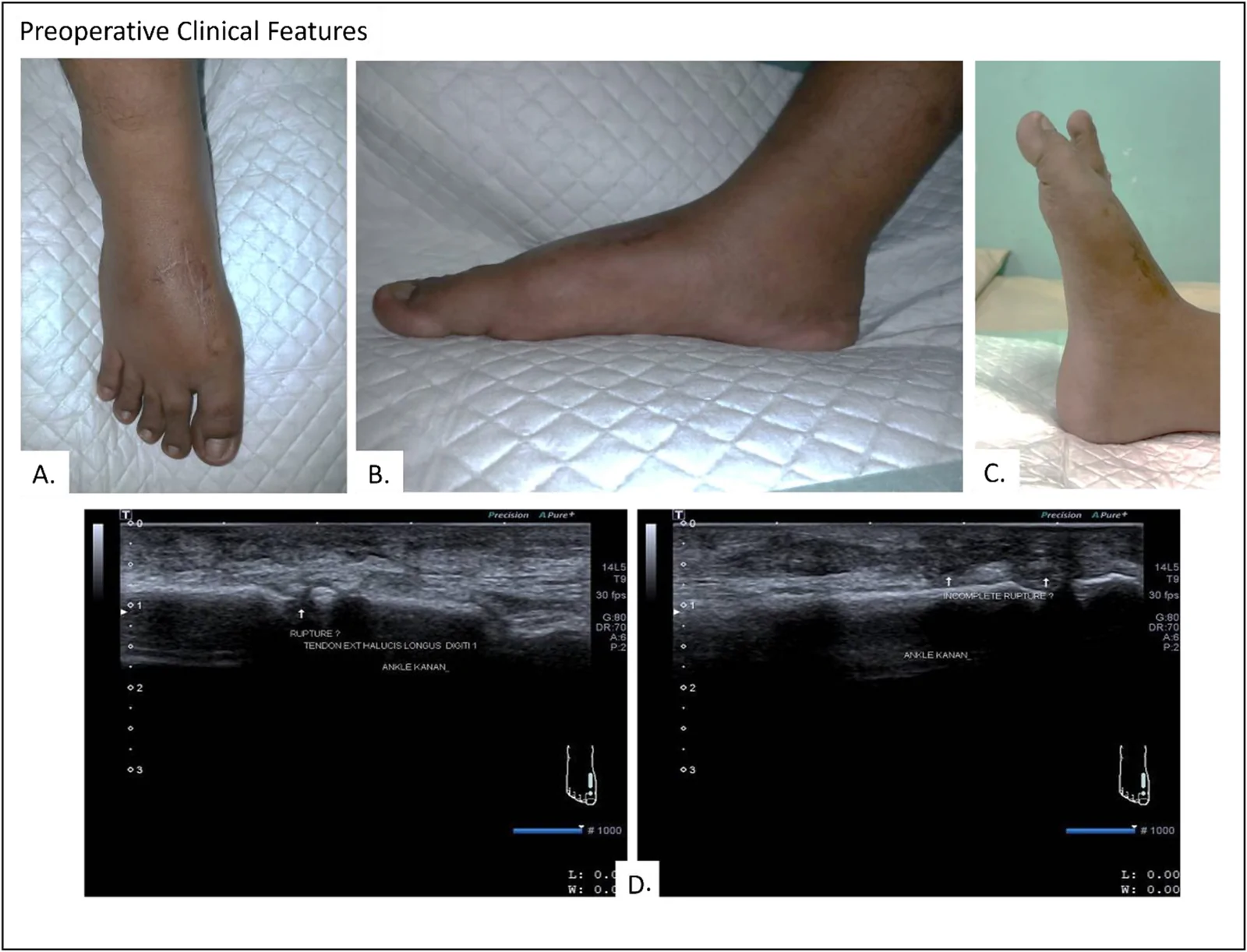
Preoperative clinical images: (A) Dorsal aspect of the foot; (B) Lateral aspect of the foot; (C) Lateral aspect of the foot with a dropped great toe; (D) Ultrasound evaluation of the EHL rupture.

A 34-year-old man presented to our outpatient clinic with the chief complaint of persistent pain in his right great toe for 12 months. He had a history of a laceration of the zone-4 dorsal aspect of his foot (Fig. 1) due to a grinding-machine injury and underwent direct surgical repair within 24 hours post-injury. Following clinical evaluation 6 weeks after the first surgery, the patient complained of persistent pain and inability to extend his great toe. Ultrasound evaluation showed rupture of the EHL with a gap and fibrotic tissue surrounding the remnant (Fig. 1). The patient did not seek treatment for a year due to economic burden (Table 1).

**Table 1:** Timeline of the disease.

Time point	Clinical event or findings	Diagnostic assessment
Initial injury	Laceration of zone-4 dorsal part of the foot due to grinding machine injury	
24 hours after injury	Direct surgical repair	
6 weeks after injury	- Persistent pain	Ultrasound evaluation showed EHL rupture with gap and fibrotic tissue surrounding the remnant
	- Inability to extend great toe	
5 months after injury	Referred to Dr. Soetomo General Academic Hospital Orthopaedic and Traumatology Outpatient Clinic	
Lost to follow-up due to economic burden		
12 months after injury	- Admitted for surgery	Intraoperative exploration result:
	- Preoperative AOFAS score: 42/100	- EHL rupture with fibrotic tissue filling the gap
	- Treated with open exploration, remnant freshening, adhesiolysis, and autologous tendon grafting with ITB	
		- The proximal stump was found at the level of the extensor retinaculum
		- After freshening, a 10 cm gap was identified between healthy proximal and distal stumps
Immediately after surgery	- The reconstructed tendon showed good tension and gliding	
	- Application of below knee cast with plantigrade position and the great toe held on neutral position	
	- Immobilized for 2 weeks	
2 weeks after surgery	- Start of passive ROM exercise of interphalangeal and metatarsophalangeal joint movement	
	- AOFAS score: 65/100	
3 weeks after surgery	- Pain was starting to be able to be tolerated	
	- Evaluation of metatarsophalangeal and interphalangeal joint extension and flexion ROM	
3 months after surgery	- Able to fully move great toe	
	- Able to directly return to work	
	- AOFAS score: 100/100	

**Figure 2.**
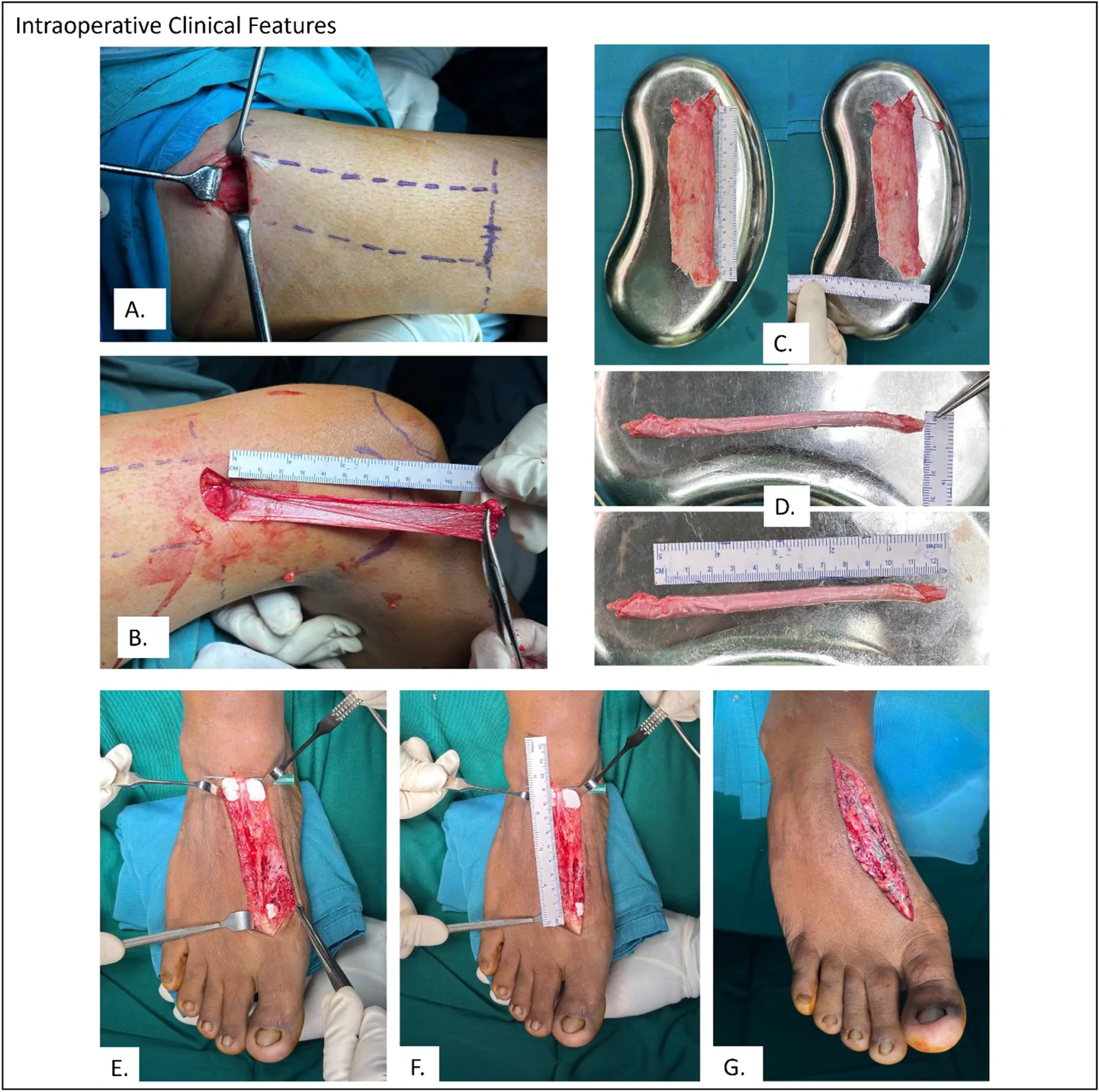
Intraoperative iliotibial band harvest: (A) Double horizontal incisions for exposure of the proximal and distal parts of the iliotibial band; (B) Harvested iliotibial band stripped and removed through the distal incision; (C) Harvested iliotibial band graft measuring 12 cm × 4 cm; (D) After tubularization, the graft measures 4 mm in diameter and 13 cm in length; (E) EHL rupture gap with remnant after exploration; (F) Gap of 10 cm after freshening of the distal and proximal stumps; (G) Graft after being sutured with a Pulvertaft double-weave technique to the proximal and distal stumps of the EHL.

The patient was admitted for surgery 12 months after the incident and treated with open exploration, freshening of the remnant, adhesiolysis, and autologous tendon grafting with ITB (Fig. 2). Intraoperative evaluation showed that there was a 10 cm gap between the healthy distal and proximal stumps, and fibrotic tissue was filling the gap (Fig. 2). Upon exploration, the proximal stump was located on the extensor retinaculum. We harvested the ITB through two horizontal incisions approximately 2 cm in width at the mid femur and supracondylar femur levels, obtained a graft measuring 4 × 13 cm, and then tubularized it into a 0.5 cm-diameter tendon graft (Fig. 2). The tendon graft was then sutured using a Pulvertaft double-weave technique to the healthy portions of the proximal and distal stumps and inserted beneath the inferior extensor retinaculum. The EHL injury is further classified into six zones, with this case demonstrating an injury at zone 4 (Supplemental Digital Content Image 1, available at: https://links.lww.com/IJSCR/A122)[1].

**Figure 3.**
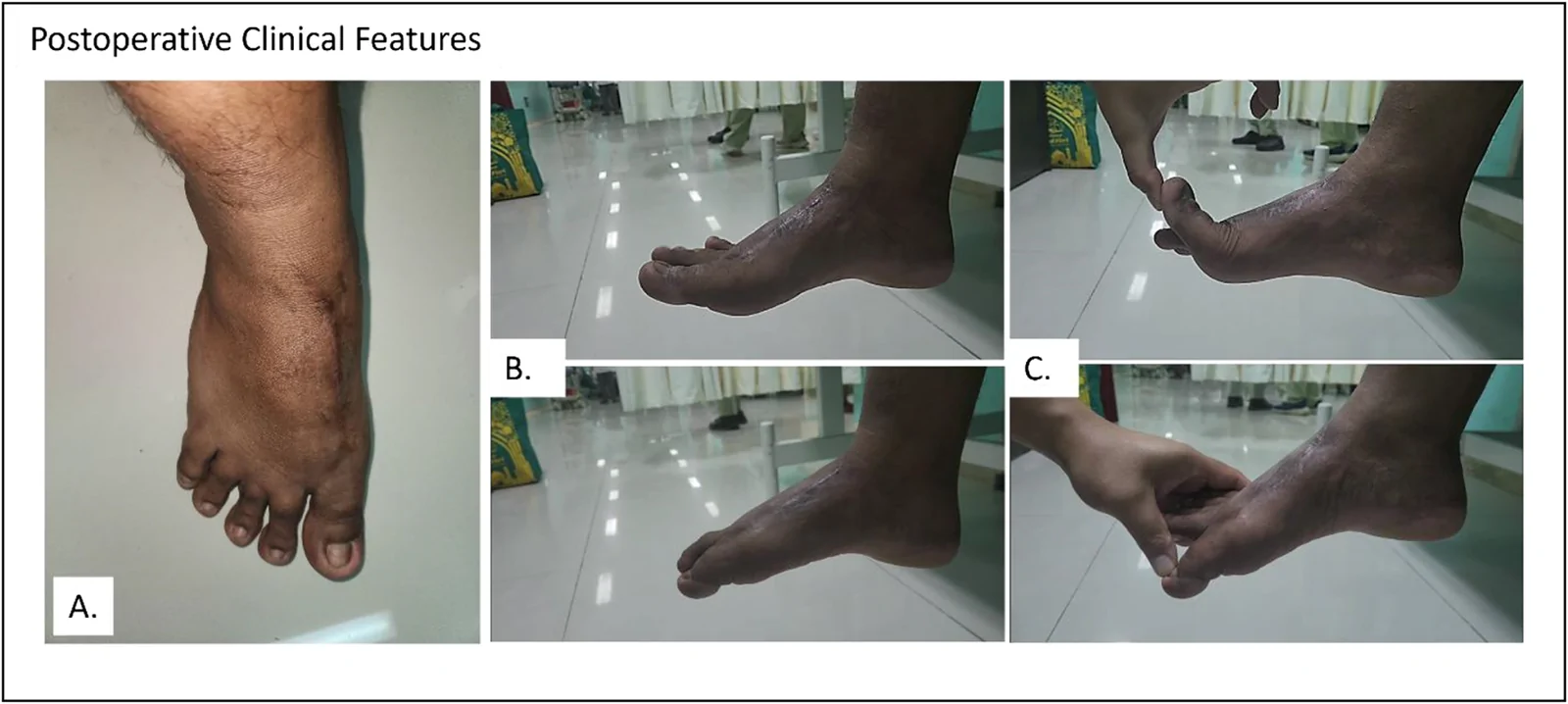
Three-month evaluation post-surgery: (A) Dorsal view of the foot postoperatively; (B) Active ROM for dorsiflexion and plantarflexion of the great toe; (C) Passive ROM for dorsiflexion and plantarflexion of the great toe.

The evaluation directly after tendon reconstruction using ITB shows that there is no lag, and the gliding within the extensor retinaculum is good. The patient is then applied a below-knee cast in a plantigrade position, and his great toe is held in neutral position with tape applied to the plantar side of the great toe to prevent interphalangeal flexion (Supplemental Digital Content Image 2, available at: https://links.lww.com/IJSCR/A122). The patient was immobilized for 2 weeks, then started early passive ROM exercises of his interphalangeal and metatarsophalangeal joints, and was able to fully move his great toe at the third month post-surgery (Fig. 3). The function of toe dorsiflexion was restored, with the patient able to move through 0–60° of passive ROM and 0–50° of active ROM. The American Orthopaedic Foot and Ankle Society (AOFAS) score evaluation preoperatively, 2 weeks postoperatively, and 3 months postoperatively shows a significant increase to 42/100, 65/100, and 100/100, respectively (Supplemental Digital Content Image 3, available at: https://links.lww.com/IJSCR/A122).

### Clinical findings

Immediately after the injury, the patient was unable to extend his right great toe, thus raising a high clinical suspicion of EHL rupture. The previous primary repair for this patient failed, and there was a gap with fibrotic tissue between the proximal and distal stumps, which was demonstrated by ultrasound evaluation 6 weeks post-trauma and confirmed intraoperatively. Following tendon grafting, with good tensioning, the Pulvertaft double-weave technique demonstrated a good clinical outcome.

### Diagnostic assessment

The diagnostic methods in this case report are clinical evaluation and ultrasound evaluation. The challenges of ultrasound evaluation depend on the expertise and experience of the user.

### Therapeutic intervention

The therapeutic intervention in this study is surgical, treating the related clinical condition operatively with an autologous graft using the ITB. The novelty of this research is the use of a tubularized fascia structure (harvested from the ITB) sutured end to end to the proximal and distal stumps of the remnant using a Pulvertaft double-weave suture technique. The patient consented to the surgical procedure as well as follow-up evaluation for publication. The intervention was performed by an orthopaedic surgeon with 20 years of orthopedic experience. No complications occurred during this therapeutic intervention or in the several months after the procedure.

### Follow-up and outcomes

The patient’s clinical condition was monitored and evaluated after removal from temporary immobilization in a short below-knee cast. We evaluated passive range of motion (ROM) for flexion and extension of his metatarsophalangeal and interphalangeal joints, as well as active ROM exercises as tolerated, starting at 3 weeks postoperatively.

At 2 weeks and 3 months post-surgery, this patient was evaluated by measuring the ROM for flexion and extension of his metatarsophalangeal and interphalangeal joints, and by the AOFAS score. The patient stated that there were no complaints regarding the postoperative results, as he was able to return to work 3 months after the surgery.

## Discussion

Our treatment shows that a surgical technique for EHL reconstruction using an autologous graft from the ITB has proven to be reliable and effective for long-term functional results. The patient had a previous injury to the zone-4 dorsum of the foot. The most common treatments for this type of injury are direct repair, tendon transfer, tendon lengthening, tendon grafting from other sites (commonly using the hamstring, semitendinosus, or peroneus longus), or scar tissue reconstruction^[^2,6–9^]^.

Currently, the ITB is increasingly popular for anterior cruciate ligament (ACL) reconstruction, especially in pediatric population, in which its use in postoperative evaluation shows good functional recovery of the knee extensor mechanism^[^10,11^]^. The use of the ITB is chosen over the peroneus longus specifically in an ACL procedure that requires added rotational control via a lateral extra-articular procedure, as the peroneus longus does not serve the anatomical and clinical purpose of extra-articular reinforcement. A previous cadaveric study reported that the biomechanical characteristics and elasticity of the gracilis tendon, whether proximal or distal, and the anterolateral ligament (ALL) were all lower than those of the ITB, and the ITB’s Young’s modulus is comparable to that of the ALL; therefore, their stiffness is almost the same^[^12,13^]^ Harvesting the ITB itself has its own recommended technique, in which the incision should be made 5 cm proximal to the articular surface of the lateral femoral condyle or 13 mm proximal to the lateral femoral epicondyle, and the graft should be less than 21 cm, as a longer graft may contribute to tensor fascia lata rupture[14].

We used the Pulvertaft double-weave fixation technique in this treatment for tendon fixation. Previous reports have shown good results after reconstruction with the Pulvertaft double-weave technique to reconstruct the EHL with the EDL III^[^15,16^]^. The difference between this case and the previously described technique is the use of fixation, in that in this case we used external support with a short-leg cast. The patient then underwent rehabilitation and physiotherapy using several steps involving pain management and edema reduction, improving ankle mobility, and improving the strength of intrinsic and extrinsic muscles[16].

It is necessary to temporarily immobilize this patient to prevent the complication of re-rupture, as occurred with the previous direct repair attempt in this patient. The use of a graft is preferable in this case due to the long gap, and our repair using multi-level sutures with a Pulvertaft double-weave technique has been shown to increase strength in the management of this type of injury[15]. This case report can be further expanded by increasing the number of cases to form a case series and may be useful for future recommendations for clinical practice regarding long-gap EHL tendon rupture or neglected EHL tendon rupture. This case report has been prepared in line with the SCARE checklist[17].

## Conclusion

Surgical treatment for acute or neglected cases of EHL tendon rupture is necessary. Outcomes are variable, largely because of the risk of tendon re-rupture from inappropriate postoperative immobilization. An ITB graft provides a novel autologous tendon graft technique for reconstruction of the EHL, with good clinical outcomes when combined with the Pulvertaft double-weave tendon suture technique and proper immobilization.

## Data Availability

All data relevant to the case are included in the article

## Research registration unique identifying number (UIN)

This is not a First-in-Man case report. The intervention described has been previously reported in the literature.
